# A circular RNA derived from PLXNB2 as a valuable predictor of the prognosis of patients with acute myeloid leukaemia

**DOI:** 10.1186/s12967-021-02793-7

**Published:** 2021-03-23

**Authors:** Leilei Lin, Yu Wang, Sicheng Bian, Lili Sun, Zhibo Guo, Desheng Kong, Linlin Zhao, Dan Guo, Qi Li, Min Wu, Yuhuang Wang, Yuying Wang, Yinghua Li

**Affiliations:** 1 Department of Hematology, The First Affiliated Hospital, Harbin Medical University, 23 Youzheng Street, Nan Gang District, Harbin, 150001 China; 2 Department of Hematology, The Fourth Affiliated Hospital, Harbin Medical University, 37 Yiyuan Street, Nan Gang District, Harbin, 150001 China; 3 Department of Blood Transfusion, The First Affiliated Hospital, Harbin Medical University, 23 Youzheng Street, Nan Gang District, Harbin, 150001 China

**Keywords:** Acute myeloid leukaemia, Extramedullary infiltration, Tumour biomarker, PLXNB2, CircPLXNB2

## Abstract

**Background:**

As a common haematological malignancy, acute myeloid leukaemia (AML), particularly with extramedullary infiltration (EMI), often results in a high mortality rate and poor prognosis. Circular RNAs (circRNAs) regulate biological and pathogenic processes, suggesting a potential role in AML. We have previously described the overall alterations in circRNAs and their regulatory networks between patients with AML presenting with and without EMI. This study aims to find new prognostic and therapeutic targets potentially associated with AML.

**Methods:**

qRT-PCR was performed on samples from 40 patients with AML and 15 healthy controls. The possibility of using circPLXNB2 (circRNA derived from PLXNB2) as a diagnostic and prognostic biomarker for AML was analysed with multiple statistical methods. In vitro, the function of circPLXNB2 was studied by lentivirus transfection, CCK-8 assays, flow cytometry, and Transwell experiments. Western blotting and qRT-PCR were performed to detect the expression of related proteins and genes. The distribution of circPLXNB2 in cells was observed using RNA fluorescence in situ hybridization (RNA-FISH). We also investigated the role of circPLXNB2 by establishing AML xenograft models in NOD/SCID mice.

**Results:**

By analysing the results of qRT-PCR detection of clinical samples, the expression of the circPLXNB2 and PLXNB2 mRNAs were significantly increased in patients with AML, more specifically in patients with AML presenting with EMI. High circPLXNB2 expression was associated with an obviously shorter overall survival and leukaemia-free survival of patients with AML. The circPLXNB2 expression was positively correlated with PLXNB2 mRNA expression, as evidenced by Pearson’s correlation analysis. RNA-FISH revealed that circPLXNB2 is mainly located in the nucleus. In vitro and in vivo, circPLXNB2 promoted cell proliferation and migration and inhibited apoptosis. Notably, circPLXNB2 also increased the expression of PLXNB2, BCL2 and cyclin D1, and reduced the expression of BAX.

**Conclusion:**

In summary, we validated the high expression of circPLXNB2 and PLXNB2 in patients with AML. Elevated circPLXNB2 levels were associated with poor clinical outcomes in patients with AML. Importantly, circPLXNB2 accelerated tumour growth and progression, possibly by regulating PLXNB2 expression. Our study highlights the potential of circPLXNB2 as a new prognostic predictor and therapeutic target for AML in the future.

**Supplementary Information:**

The online version contains supplementary material available at 10.1186/s12967-021-02793-7.

## Background

As the most common myeloid hematopoietic stem/progenitor cell clonal disease in adults, acute myeloid leukaemia (AML) is highly malignant [1, 2]. Aggressive leukaemia cells from the bone marrow (BM) can spread to peripheral blood or organs other than the BM, causing extramedullary infiltration (EMI) [3]. Patients with AML presenting with EMI tend to have a poor prognosis, and most of these patients are prone to relapse or have a refractory disease [4]. Despite progress in AML diagnosis and treatment regimens, more than half of patients experience short-term recurrence or reduced treatment sensitivity [5], and approximately 80% of patients with AML die [6]. Hence, the identification of new prognostic and therapeutic targets for AML is urgently needed.

Circular RNAs (circRNAs) are produced by back splicing and play a crucial role in both physiological and pathological processes [7–9], particularly cancer [10, 11]. Based on accumulating evidence, circRNAs function through the following mechanisms: regulating gene expression in *cis* at the transcriptional level [12], acting as a microRNA “sponge” and inhibiting its function [13], interacting with RNA binding proteins that regulate transcription [14], encoding peptides [15], and other functions. Due to their special covalently closed circular structure, circRNAs are strongly resistant to RNase R, are more stable than mRNAs and are often regarded as potential biomarkers for cancer [16, 17]. CircRNAs are closely related to myeloid malignancies. For example, circRNA-DLEU2 inhibits miR-496 and upregulates PRKACB expression, thus accelerating AML progression [18]; a circRNA derived from MYBL2 promotes FMS-like tyrosine kinase-3 translation by increasing polypyrimidine tract binding protein 1 expression, leading to a poor prognosis [19]; circASXL1-1-mediated regulation of BAP1 deubiquitinase activity might be a promising therapeutic option for myeloid leukaemia [20]. Our previous studies also confirmed that circRNAs may play an important role in regulating intercellular crosstalk in EMI, and we also found that PLXNB2 predicted a poor prognosis of patients with AML and might be involved in EMI regulation [21].

PLXNB2, a member of the B class of plexins, is a transmembrane receptor involved in cell migration and axonal guidance [22], regulates the cellular immune response [23, 24], functions as the receptor for angiogenin to regulate angiogenesis in malignant tumours, including AML [25], and exerts important effects on the activation of RHOA [22]. At present, high PLXNB2 expression has been confirmed in various malignant tumours, including malignant glioma, ovarian cancer and breast cancer, and is closely related to the proliferation, invasion and poor prognosis of tumours [26–28]. PLXNB2 expression is regulated by microRNAs [27, 29], but few studies have assessed the relation between PLXNB2 and circRNAs. The role of circPLXNB2 derived from the PLXNB2 gene has rarely been studied in cancer, let alone in AML.

In this study, our aims were to detect and analyse the expression of circPLXNB2 in AML patients presenting with or without EMI and healthy controls, to explore the correlation with the expression of the PLXNB2 gene, and to investigate the potential of circPLXNB2 as a new biomarker for the prognosis and treatment of AML.

## Methods

### Patient samples

We evaluated samples from 40 adults with AML presenting with or without EMI (EMI = 24 and non-EMI = 16) collected at the primary diagnosis and samples collected from 15 healthy volunteers at the Department of Hematology of the First Affiliated Hospital of Harbin Medical University. The research study was approved by the Ethics Committee of Human Experimentation at the First Affiliated Hospital of Harbin Medical University. Informed consent was based on the Declaration of Helsinki. Patients were diagnosed with AML and classified based on the standard diagnostic criteria of the French–American–British (FAB) and World Health Organization [30, 31]. Samples from patients receiving chemotherapy before collection were excluded. Bone marrow mononuclear cells (BMMCs) were obtained using a previously reported method [21]. Additional file 2: Table S1 shows our summary of the major laboratory and clinical characteristics of patients with AML.

### Cell culture

In this study, we used the human THP1, MV4;11, OCI-AML3 and HL-60 cell lines. We purchased the human AML cell lines from the Shanghai Cell Bank, Chinese Academy of Sciences. The cells were cultured in a humidified 5% CO_2_ incubator at a constant temperature of 37 °C. RPMI-1640 medium (Gibco BRL, Gaithersburg, MD, USA) was mixed with 10% foetal bovine serum (FBS, Cat. 04-001-1ACS; BI, USA), 100 U/mL penicillin and 100 U/mL streptomycin. HL-60 and OCI-AML3 cells were transfected with circPLXNB2 overexpression (circPLXNB2 OE) plasmids or circPLXNB2 short hairpin RNA (circPLXNB2 shRNA) and then harvested.

### RNA extraction and Real‐time quantitative polymerase chain reaction (qRT-PCR)

Total RNA was isolated using TRIzol reagent (Invitrogen, Carlsbad, CA, USA) according to the manufacturer’s instructions. We performed reverse transcription using Super M-MLV reverse transcriptase (BioTeke, Beijing, China) to obtain cDNA templates. qRT-PCR was performed using an Exicycler 96 sequence detection system (Bioneer, Taejon, South Korea). The primer sequences for qRT-PCR are shown in Table 1. The primers were provided by GenScript (Nanjing, China). Glyceraldehyde 3-phosphate dehydrogenase (GAPDH) was used as an internal control.

**Table 1:** Primers used for qRT-PCR

Gene	Primer Sequence (5′-3′)
circPLXNB2-F	CCCACGTCAAAGGTCAAG
circPLXNB2-R	TCTCCTCCTCCCATCTCG
has_circ_0004520-F	TGAGACCAAGAAGGAGAAAAGAAAA
has_circ_0004520-R	CTCGCACATCGAAGAGGTCA
PLXNB2-F	GCATCCGCATCACCATCC
PLXNB2-R	GGCCCAGTTTCCCGAAGA
GAPDH-F	GACCTGACCTGCCGTCTAG
GAPDH-R	AGGAGTGGGTGTCGCTGT

*F* Forward primer, *R* Reversed primer, *GAPDH* glyceraldehyde 3-phosphate dehydrogenase

### Actinomycin D assay

To block transcription, AML cells were exposed to 2 μg/mL actinomycin D (Cat. HY-17559; MCE, Shanghai, China). Then, the cells were collected, and the expression of circPLXNB2 and the PLXNB2 mRNA was detected at 4, 8, 12 and 24 h using qRT-PCR to analyse the stability of circPLXNB2.

### Construction and transfection of circPLXNB2 overexpression and knockdown vector

In brief, the human circPLXNB2 (circBase: hsa_circ_0001257, http://www.circbase.org) gene was amplified from cDNA library. The pLC5-ciR plasmid (Cat.GS0108; Geneseed Biotech, Guangzhou, China) was used to establish the recombinant circPLXNB2 overexpression lentiviral vector. The cyclization mediated sequence is introduced to effectively ensure the accurate cyclization of the linear sequence of the target circRNA. We digested the plasmids with restriction endonucleases EcoRI and BamHI (Promega, Beijing, China) and purified the fragments. After purification, T4 DNA ligase was used for ligation. The circPLXNB2 RNAi target sequences were designed by Shanghai GenePharma Co., Ltd. The sequences of circPLXNB2 shRNA and the negative control (NC) shRNA are shown in Table 2. The sequences of circPLXNB2 shRNA and shRNA NC were synthesized and then inserted into the lentiviral vector pLKO.1-EGFP-Puro (Cat.FH1717; Fenghui Biotech, Changsha, China) at the AgeI and EcoRI restriction sites (Promega, Beijing, China).

**Table 2:** Sequences of short hairpin RNAs

Gene	Sequence (5′-3′)
circPLXNB2 shRNA-1	CATCCTGGGATCCTATGTCTG
circPLXNB2 shRNA-2	ACATCCTGGGATCCTATGTCT
circPLXNB2 shRNA-3	GACATCCTGGGATCCTATGTC
shRNA-NC	GATCATACGTGCGATCAGA

*shRNA* short hairpin RNAs, *NC* negative control. CircPLXNB2 shRNA-1 was confirmed to have the best interference effect by qRT-PCR, so this sequence was selected to construct circPLXNB2 knockdown lentivirus vector

For lentiviral packaging, core and packaging plasmids were transfected into 293 T cells for 48 h before the supernatant was obtained. The lentiviral vectors were harvested and used to infect AML cells (5 × 10^5^ cells/mL); polybrene (6 μg/mL, Cat. HY-112735; MCE, Shanghai, China) was added to facilitate infection. Infection was performed at 37 °C for 24 h. After infection, we cultured the cells for 72 h in fresh complete medium. Then, the transduced AML cells were selected with puromycin. The vector of circPLXNB2 knockdown or overexpression was validated through sequencing, and the cyclization of circPLXNB2 was verified by qPCR.

### RNA fluorescence in situ hybridization (RNA-FISH)

The location of circPLXNB2 in AML cell lines was measured using a Cy3-labeled circPLXNB2 probe obtained from Shanghai GenePharma Co., Ltd. The probe sequence was as follows: 5′-CTCCTCCTCCCATCTCGTGGCCCAGGATGTCTGGGCCCCG-3′. Hybridization was performed according to the manufacturer’s instructions provided with the Fluorescent In Situ Hybridization Kit (GenePharma, Shanghai, China). The nuclei were counterstained with 4,6-diamidino-2-phenylindole (DAPI). The images were observed and captured using a Nikon inverted fluorescence microscope. We used ImageJ v1.51 software to analyse 3 separate areas in each image and quantify the nuclear and cytoplasmic levels of circPLXNB2; a detailed description of the analytical method is provided in a previous study [32].

### Cell proliferation and flow cytometry

The Cell Counting Kit-8 (CCK-8) method (Sigma-Aldrich, St. Louis, MO, USA) was used to measure cell proliferation. The analysis of the cell cycle and apoptosis was detected using a FACSCalibur flow cytometer (Becton Dickinson, Franklin Lakes, NJ, USA). For the cell cycle analysis, propidium iodide (PI, Sigma-Aldrich) was used to stain the cells. According to the manufacturer's instructions, Annexin V-FITC /PI (Sigma-Aldrich) was used for apoptotic analysis. The CCK-8 and flow cytometry (FCM) assays were performed as previously described [33].

### Transwell assays

In short, 1 × 10^5^ AML cells were cultured in the upper chamber (Corning, NY, USA) with 200 μL serum-free medium. In the subchamber, we added 800 μL of medium supplemented with 10% FBS. After 24 h, the cells in the lower compartment were collected, suspended and stained with trypan blue (Sigma-Aldrich, St. Louis, MO, USA). The numbers of cells in the subchamber were confirmed using a microscope (OLYMPUS, Tokyo, Japan).

### Western blot analysis

Proteins were separated through using sodium dodecyl sulfate polyacrylamide gel electrophoresis, and transferred to polyvinylidene fluoride membranes. We used 5% skim milk powder to block the membranes. The membranes were incubated overnight at 4 °C with primary antibodies against the following proteins: PLXNB2 (Cat. ab229950; Abcam, Shanghai, China), cyclin D1(Cat. ab16663; Abcam, Shanghai, China), B-cell lymphoma-2 (BCL2, Cat. ab182858; Abcam, Shanghai, China), and BCL2-associated X protein (BAX, Cat. ab32503; Abcam, Shanghai, China). We used the anti-β-actin antibody (Cat. ZRB1312; Sigma, St. Louis, MO, USA) as the loading control. Bands on the Western blots were visualized using the BCIP/NBT kit (Sigma-Aldrich, St. Louis, MO, USA). Image analysis software (ImageQuant TL; Amersham Biosciences, USA) was used to quantify the band intensities on the immunoblots.

### Tumor xenograft assay

The animal study was approved by the Animal Ethics Committee of Harbin Medical University. We conducted all experiments in accordance with the institutional animal care procedures. Twenty-four female NOD/SCID mice aged 6–8 weeks, weighing 17.85 ± 0.65 g, were purchased from the Beijing Hua Fu Kang Biotechnology Co., Ltd. The mice were randomly divided into four groups (n = 6 mice/group) and housed under specific pathogen-free conditions. Each mouse received a subcutaneous injection of AML cells (1 × 10 ^7^ in 100 μL of PBS) in the right flank. The four groups were injected with different cell preparations: OCI-AML3 shRNA NC cells, OCI-AML3 circPLXNB2 shRNA cells, HL-60 circPLXNB2 OE cells, and HL-60 vector cells. During 28 days of routine feeding, the tumour volumes were measured twice a week. At the end of the 28-day observation period, all mice were sacrificed by cervical dislocation, and tumour specimens were collected, imaged, and measured for size and weight. TUNEL staining, haematoxylin and eosin (H&E) staining, qRT-PCR, immunohistochemistry, and Western blotting were also performed on tumour samples from 3 mice in each group.

### Haematoxylin and eosin staining

The xenograft tissue was immobilized with paraformaldehyde at a concentration of 4%, embedded in paraffin, and then cut into thin 5-µm sections. After dewaxing, the sections were rehydrated with a graded series of ethanol solutions and then stained with standard H&E. We observed the results using a microscope (OLYMPUS, Tokyo, Japan) at 200× magnification and obtained images using a microscope photo system (OLYMPUS, Tokyo, Japan).

### TUNEL assay

According to the manufacturer’s instructions, the numbers of apoptotic cells in mouse tissues were estimated with TUNEL staining using an In Situ Apoptosis Detection Kit (Cat. ab206386; Abcam, Cambridge, UK). Three areas were evaluated from each biopsy. Apoptotic cells in the biopsies were stained brown. We observed the results using a microscope (OLYMPUS, Tokyo, Japan) at 400× magnification and obtained images using a microscope photo system (OLYMPUS, Tokyo, Japan).

### Immunohistochemical staining

In short, tissue sections were sequentially incubated with an anti-PLXNB2 (Cat. NBP1-89620; Novus Biological, Littleton, CO, USA) antibody overnight at 4 °C and then incubated with secondary antibodies at 25 °C for one hour. We observed the results using a microscope (OLUMPUS, Tokyo, Japan) at 400× magnification and acquired images using a microscope photo system (OLUMPUS, Tokyo, Japan). Then, we used Image-Pro Plus v6.0 (Media Cybernetics, Bethesda, MD, USA) to quantitatively score tissue slices. The software measured the average optical density of the selected area, and the results indicated the expression levels of the candidate proteins in tissues.

### Statistical analyses

Analyses of all data from at least three independent experiments were performed using GraphPad Prism 5.0 (GraphPad Software, La Jolla, CA, USA). We present the data as means ± SD. Depending on the experiment, one-way analysis of variance (ANOVA) or two-way ANOVA was selected to calculate the statistical significance. When comparing the differences in categorical variables between groups of patients with AML, we used Fisher's exact test or the Chi-Square test. The Mann–Whitney U test was used to compare the differences in continuous variables between the two groups. We evaluated the diagnostic value of circPLXNB2 in distinguishing patients with AML from healthy volunteers by performing receiver operating characteristic (ROC) and area under the ROC curve (AUC) analyses. The survival rate was determined using the Kaplan–Meier method and assessed with the log-rank test. We calculated Pearson’s correlation coefficients to examine the correlation between circPLXNB2 and PLXNB2 mRNA expression. *P* < 0.05 was considered significant.

## Results

### PLXNB2 and circPLXNB2 expression were significantly increased in patients with AML

**Fig. 1:**
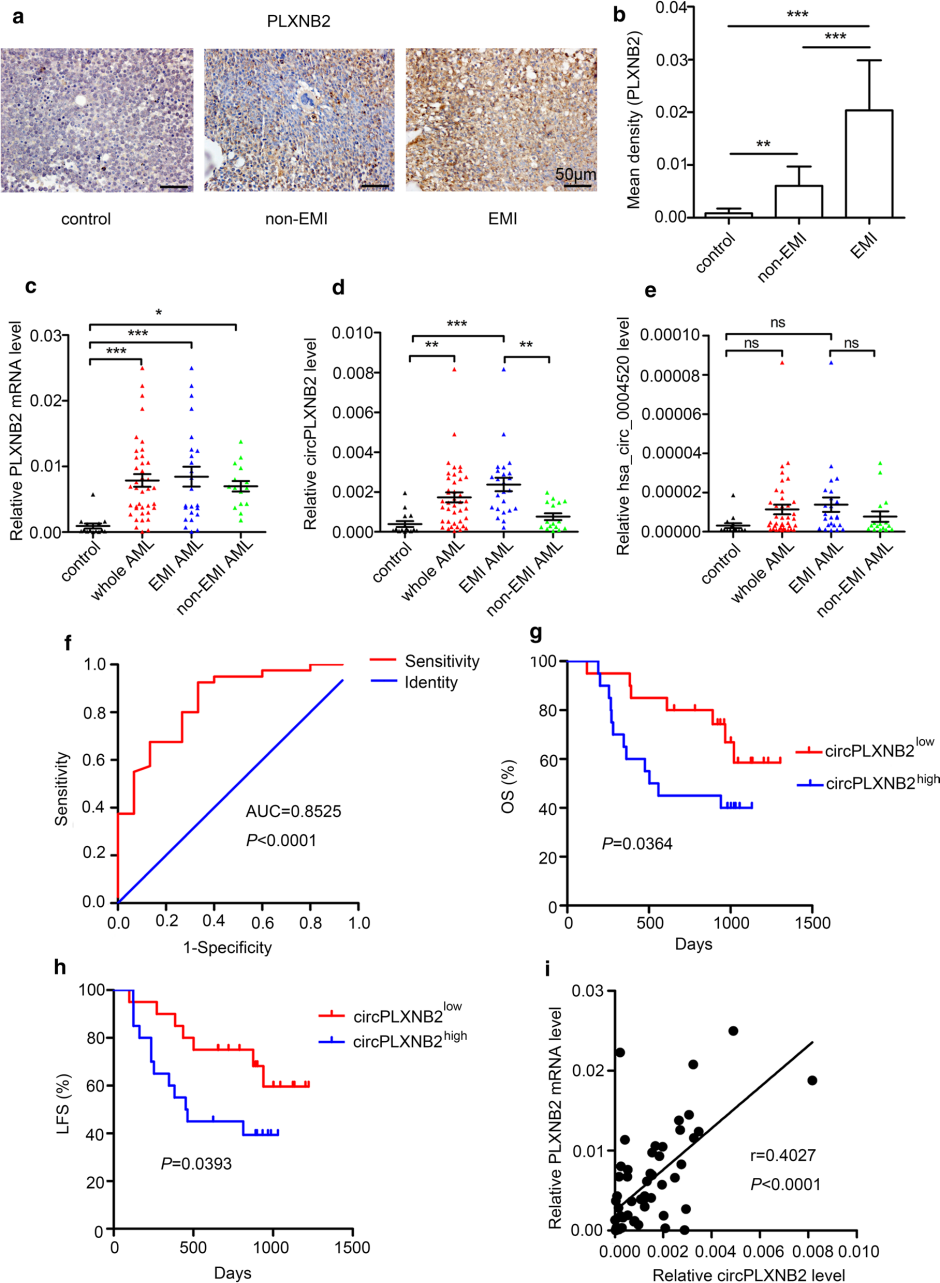
PLXNB2 and circPLXNB2 expression in AML samples and the relationship between circPLXNB2 and clinical outcomes. **a** Representative images of PLXNB2 IHC staining in samples from patients with AML and healthy controls; **b** image software was used to measure the average optical density of the selected areas in PLXNB2 IHC-stained sections from patients with AML and healthy controls. EMI, n = 4; non-EMI, n = 4; control, n = 4 samples. The original magnification was 400×. The expression of the PLXNB2 mRNA (**c**), circPLXNB2 (**d**) and hsa_circ_0004520 (**e**) in the samples was detected using qRT-PCR. Control, n = 15; EMI, n = 24; non-EMI, n = 16; whole AML = EMI + non-EMI, n = 40 samples. **f** ROC and AUC analysis of circPLXNB2 expression in 40 patients with AML. Kaplan–Meier analyses of OS (**g**) and LFS (**h**) in patients with AML stratified according to circPLXNB2 expression levels (n = 40). **i** The correlation between the expression of circPLXNB2 and the PLXNB2 mRNA was examined using Pearson’s correlation test. Each experiment was repeated three times. *P < 0.05, **P < 0.01, and ***P < 0.001. *EMI* extramedullary infiltration, *AUC* area under the ROC curve, *OS* overall survival, *LFS* Leukaemia-free survival, *ns* not significant

Whole-genome and circRNA microarrays were conducted from 8 samples of AML patients with or without EMI and 4 samples of healthy controls, which indicating PLXNB2, hsa_circ_0004520 and hsa_circ_0001257 were likely to be involved in the regulation of intercellular crosstalk associated with EMI [21]. Immunohistochemical (IHC) staining was performed on bone marrow biopsy specimens obtained from patients above showed that PLXNB2 protein expression were significantly higher in patients with AML, particularly in patients with AML presenting with EMI (Fig. 1a, b). qRT-PCR analysis of BMMCs from patients with AML presenting with or without EMI (EMI = 24, non-EMI = 16) and healthy controls (n = 15) revealed that PLXNB2 mRNA was highly expressed in patients with AML (Fig. 1c). Hsa_circ_0001257 is located on chromosome 22q13.33 and transcribed from the host PLXNB2 gene; therefore, we named this circRNA circPLXNB2. Results from the previous circRNA microarray showed that the expression of hsa_circ_0004520 and circPLXNB2 was significantly higher in patients with AML presenting with EMI than in patients with non-EMI AML (4.343 and 3.785, respectively). Pathway analysis shown that hsa_circ_0001257 and hsa_circ_0004520 both participated in pathways related to cell–cell interaction [21]. Then, qRT-PCR was used to detect the expression of these two circRNAs in samples from the patients and healthy controls. Notably, circPLXNB2 was expressed at higher levels in patients with AML presenting EMI, compared with patients with non-EMI AML (Fig. 1d). And we found that the expression of circPLXNB2 was significantly upregulated in 65% (26 of 40) of whole AML samples (Additional file 1: Figure S1). A significant difference in hsa_circ_0004520 expression was not observed between patients with EMI and without EMI (Fig. 1e). CircRNAs can function as “sponges” of miRNAs, or regulate the expression of parental genes through a certain mechanism [34, 35]. Thus, hsa_circ_0001257 (circPLXNB2) derived from parental PLXNB2 might regulate the expression of host gene promoting the EMI in AML.

### The expression of circPLXNB2 correlates with a poor prognosis for patients with AML

We analysed the potential diagnostic value of circPLXNB2 expression in differentiating individuals with AML from a healthy population using ROC curves. The AUC was 0.8525 (*P* < 0.0001, Fig. 1f), indicating that circPLXNB2 has good potential as a diagnostic biomarker. We divided the cohort of 40 AML patients into two groups: high circPLXNB2 expression (circPLXNB2^high^) group and low circPLXNB2 expression (circPLXNB2^low^) group. No obvious differences in age, sex, white blood cells count, platelet count, haemoglobin levels, FAB subtypes, karyotype classifications and mutations in six genes were observed between the circPLXNB2^high^ and circPLXNB2^low^ groups (Additional file 2: Table S1). While the expression of circPLXNB2 was correlated with clinical outcomes of AML patients. The Kaplan–Meier analysis revealed that patients with AML in the circPLXNB2^high^ group had a remarkably shorter overall survival (OS, Fig. 1g, P = 0.0364) and leukaemia-free survival (LFS, Fig. 1h, P = 0.0393) than patients with AML in the circPLXNB2^low^ group. Pearson’s correlation coefficients were calculated to investigate the correlation between the expression of circPLXNB2 and its parental gene PLXNB2 in patients with AML, and a positive relationship was identified between circPLXNB2 expression and the PLXNB2 mRNA expression (r = 0.4027, *P* < 0.0001, Fig. 1i).

### Characteristics of circPLXNB2 and its distribution in AML cells

**Fig. 2:**
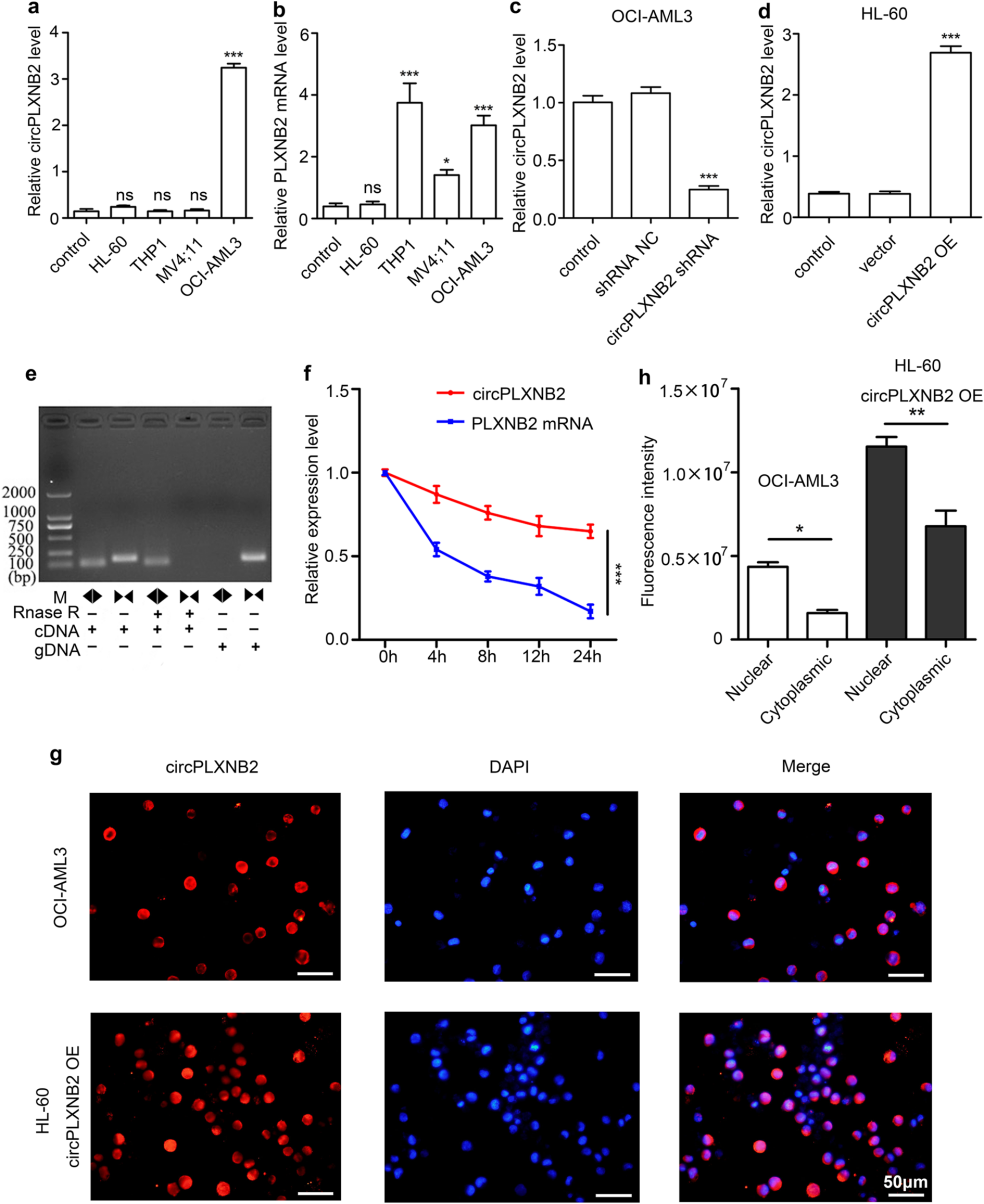
Expression of circPLXNB2 and PLXNB2 mRNA, and characterization and distribution of circPLXNB2 in AML cells. The expression of circPLXNB2 (**a**) and the PLXNB2 mRNA (**b**) in different AML cell lines was detected using qRT-PCR; BMMCs from healthy volunteers served as controls. qRT-PCR analysis was used to verify that transfection of OCI-AML3 cells (**c**) and HL-60 cells (**d**) with circPLXNB2 shRNA or shRNA NC or vector or circPLXNB2 OE plasmid was successful. **e** AML cells were treated with or without RNase R, and qRT-PCR was used to assess circPLXNB2 amplification with divergent primers and convergent primers using the template cDNA and gDNA derived from AML cells. **f** qRT-PCR assay of the expression of circPLXNB2 and the PLXNB2 mRNA in AML cells treated with the transcriptional inhibitor actinomycin D (2 μg/mL) at the indicated time points. The PLXNB2 mRNA was used as a control. **g** Identification of the circPLXNB2 distribution in OCI-AML3 cells and HL-60 circPLXNB2 OE cells using RNA-FISH. **h** Quantification of nuclear and cytoplasmic levels of circPLXNB2 staining in AML cells. Nuclei were stained with DAPI. The original magnification was 400×. Each experiment was repeated three times. *P < 0.05, **P < 0.01, and ***P < 0.001. *CircPLXNB2 OE* circPLXNB2 overexpression, *circPLXNB2 shRNA* circPLXNB2 short hairpin RNA, *NC* negative control, *DAPI* 4,6-diamidino-2-phenylindole, *cDNA* complementary DNA, *gDNA* genomic DNA, *ns* no significance

We detected the levels of circPLXNB2 and the PLXNB2 mRNA in multiple AML cell lines. The expression of circPLXNB2 and the PLXNB2 mRNA was high in OCI-AML3 cells and low in HL-60 cells (Fig. 2a, b). Using the technology of lentivirus transfection, we constructed OCI-AML3 cells expressing the circPLXNB2 shRNA and HL-60 cells overexpressing circPLXNB2. qRT-PCR was used to verify the knockdown efficiency of circPLXNB2 shRNA in OCI-AML3 cells (Fig. 2c), and the expression of circPLXNB2 was up-regulated after infection with circPLXNB2 OE into HL-60 cells (Fig. 2d).

We conducted PCR using cDNA and genomic DNA (gDNA) pretreated with or without the RNase R exonuclease to further elucidate the characteristics of circPLXNB2. CircPLXNB2 resisted degradation by RNase R, whereas convergent primers were unable to amplify the cDNA that was reverse transcribed from mRNA (Fig. 2e). Following an actinomycin D treatment, the half-life of circPLXNB2 was longer than the PLXNB2 mRNA, indicating that circPLXNB2 is highly stable (Fig. 2f). Taken together, our results indicate that circPLXNB2 is a stable circular molecule. Moreover, we performed RNA-FISH to observe the cellular distribution of circPLXNB2, and the results revealed that circPLXNB2 is mainly located in the nucleus. (Fig. 2g, h).

### CircPLXNB2 may function as an oncogene in vitro

**Fig. 3:**
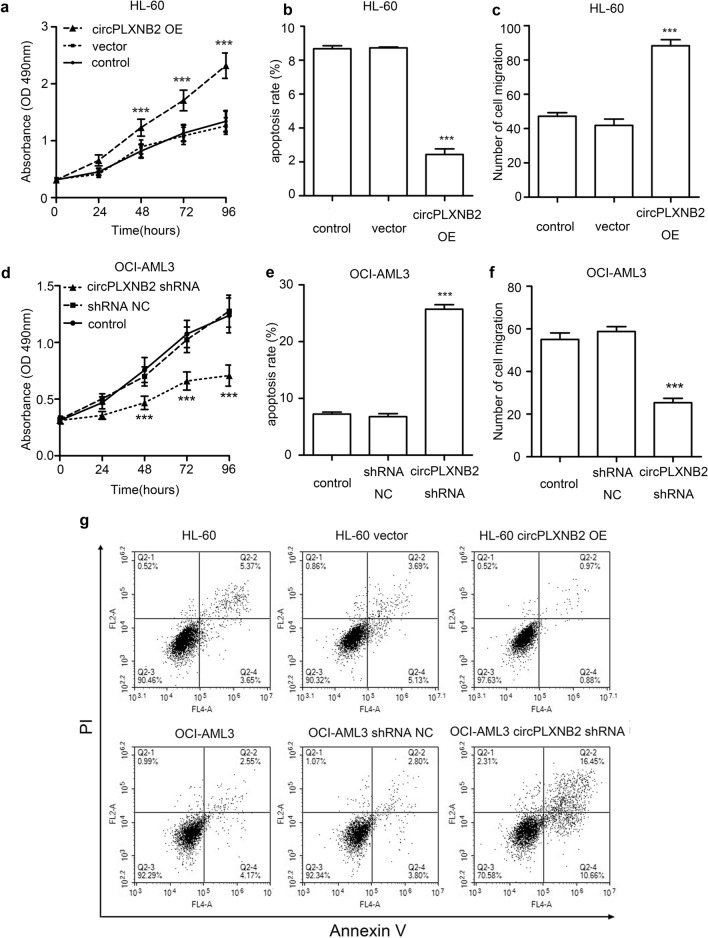
CircPLXNB2 promotes the proliferation and migration of AML cells and inhibits apoptosis in vitro. Cell proliferation was measured with the CCK-8 method. Apoptosis was analysed with FCM analysis. A Transwell assay was performed to detect cell migration. CircPLXNB2 OE significantly increased the proliferation rates (**a**) and migration (**c**) of HL-60 cells, and decreased the number (**b**) of apoptotic HL-60 cells. The circPLXNB2 shRNA significantly decreased the proliferation rates (**d**) and migration (**f**) of OCI-AML3 cells, and increased the number (**e**) of apoptotic OCI-AML3 cells. **g** Representative FCM plots of the effects of circPLXNB2 expression on apoptosis. Q2-4: early apoptotic cells; Q2-2: late apoptotic cells. The combined percentages of apoptotic cells in the Q2-4 and Q2-2 quadrants represent the percentage of total apoptotic cells. Nontransfected cells were used as a control, and the vector or shRNA NC was used as a negative control. Each experiment was repeated three times. ***P < 0.001 compared with the control. *CircPLXNB2 OE* circPLXNB2 overexpression, *circPLXNB2 shRNA* circPLXNB2 short hairpin RNA, *NC* negative control, *PI* propidium iodide

Next, we explored the oncogenic role of circPLXNB2 in AML cells. We performed CCK-8 and FCM to assess the effect of circPLXNB2 on cell proliferation and apoptosis and its proleukaemic activity in vitro. Compared with the control cells, the proliferation of circPLXNB2-overexpressing HL-60 cells was significantly increased (Fig. 3a), while the proliferation of OCI-AML3 cells expressing the circPLXNB2 shRNA was significantly decreased (Fig. 3d). In order to confirm further that circPLXNB2 was responsible for the phenotypes, we rescued the expression of circPLXNB2 in OCI-AML3 cells which had circPLXNB2 silenced (Additional file 3: Figure S2a). Result showed that forced overexpression of circPLXNB2 could significantly rescue the proliferation phenotype and promoted the cell growth (Additional file 3: Figure S2b). According to the FCM and Annexin V-FITC staining, apoptosis was inhibited in HL-60 cells transfected with the circPLXNB2 OE vector (Fig. 3b, g), while the apoptosis of OCI-AML3 cells transfected with the circPLXNB2 shRNA was increased (Fig. 3e, g). Subsequently, a Transwell assay was used to detect the migration of AML cells. The migration of circPLXNB2 OE HL-60 cells was enhanced compared with the control cells (Fig. 3c). At the same time, OCI-AML3 cells transfected with the circPLXNB2 shRNA showed reduced migration compared with untreated OCI-AML3 cells (Fig. 3f).

**Fig. 4:**
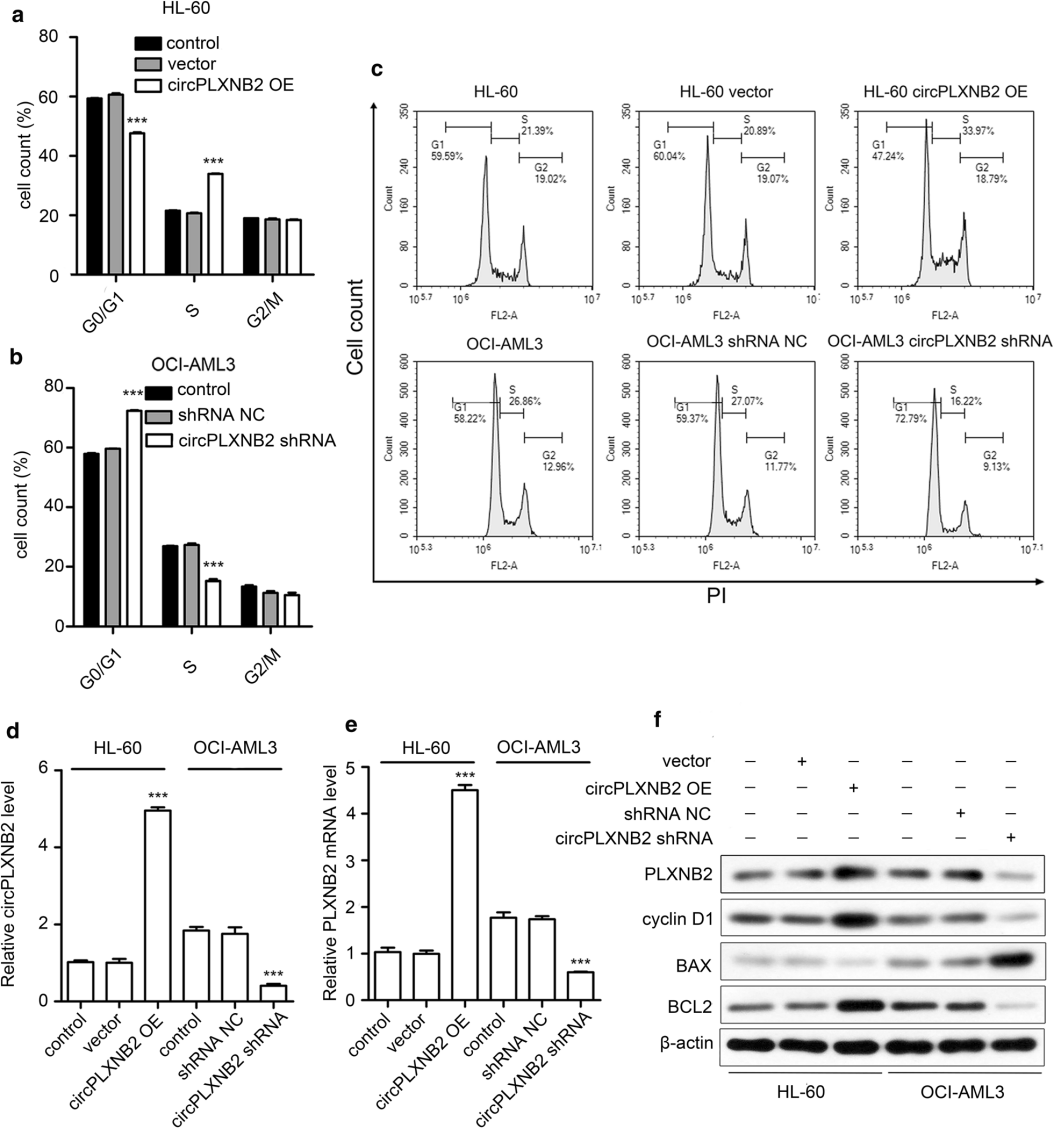
Effect of circPLXNB2 expression on the levels of PLXNB2 and cell cycle-related proteins. **a** CircPLXNB2 OE significantly decreased the proportion of HL-60 cells in G0/G1 phase and increased the proportion of HL-60 cells in S phase. **b** The circPLXNB2 shRNA significantly increased the proportion of OCI-AML3 cells in G0/G1 phase and decreased the proportion of OCI-AML3 cells in S phase. **c** Representative FCM plots of the effects of circPLXNB2 expression on cell cycle progression. The expression of circPLXNB2 (**d**) and the PLXNB2 mRNA (**e**) was detected using qRT-PCR. **f** Representative Western blots showing the expression of BCL2, BAX, cyclin D1 and PLXNB2 in AML cells; β-actin was used as a loading control. Nontransfected cells were used as a control, and the vector or shRNA NC was used as a negative control. Each experiment was repeated three times. ***P < 0.001 compared with the control. *CircPLXNB2 OE* circPLXNB2 overexpression, *circPLXNB2 shRNA* circPLXNB2 short hairpin RNA, *NC* negative control

We detected a decrease in the proportion of circPLXNB2 OE HL-60 cells in G0/G1 phase and an increased proportion in S phase (Fig. 4a, c). Moreover, compared with controls, the proportion of OCI-AML3 cells transfected with the circPLXNB2 shRNA in G0/G1 phase was increased, and the proportion of cells in S phase was decreased (Fig. 4b, c). We performed qRT-PCR to detect the expression of circPLXNB2 and the PLXNB2 mRNA, and used Western blotting to assess the levels of the PLXNB2 protein and other proteins associated with the cell cycle or apoptosis. The experimental results confirmed higher expression of the PLXNB2 mRNA and protein in AML cells with high circPLXNB2 expression than in AML cell lines with low expression levels of circPLXNB2 (Fig. 4d–f). Moreover, after the circPLXNB2 expression was rescued in OCI-AML3 cells which had circPLXNB2 silenced, the expression level of PLXNB2 mRNA was also significantly increased (Additional file 3: Figure S2c). BAX expression was decreased, while the expression of cyclin D1 and BCL2 was increased in HL-60 cells overexpressing circPLXNB2 (Fig. 4f). Additionally, the circPLXNB2 shRNA increased the level of BAX expression and decreased the expression of cyclin D1 and BCL2 (Fig. 4f).

### CircPLXNB2 increases the leukemic burden in AML xenograft tumour models

**Fig. 5:**
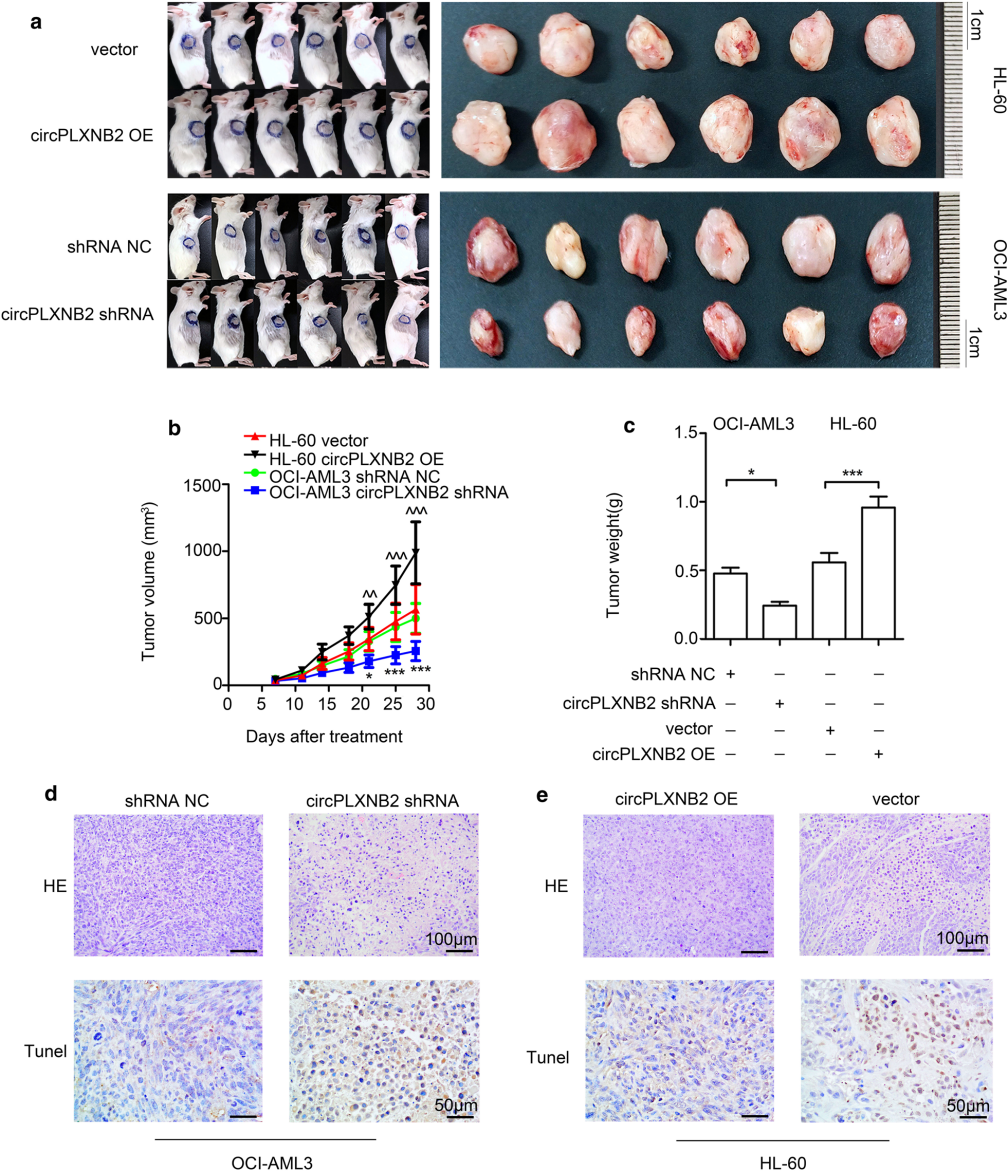
CircPLXNB2 promoted tumour growth in AML xenograft models. **a** Images of tumour-bearing mice show that the tumours were larger in circPLXNB2 OE-treated mice and smaller in circPLXNB2 shRNA-treated mice. The tumour sizes (**b**) were observed for 28 days, the mice were sacrificed at the end of 28 days, and the weights (**c**) of subcutaneous tumours were also measured after 28 days. H&E and TUNEL staining of xenograft tumour tissues obtained from three mice randomly selected from each group (n = 6). Representative fields are shown. **d** H&E (upper panel) and TUNEL (lower panel) staining of the xenograft tumour tissues from mice treated with OCI-AML3 cells transfected with shRNA NC or circPLXNB2 shRNA. **e** H&E (upper panel) and TUNEL (lower panel) staining of the xenograft tumour tissues from mice treated with HL-60 cells transfected with vector or circPLXNB2 OE. In the H&E-stained sections, the original magnification was 200×. Positive cells are indicated by brown staining in the TUNEL-stained sections, with an original magnification of 400×. All parameters were measured in three separate experiments. The vector or shRNA NC was used as a control. *P < 0.05, ***P < 0.001, ^^*P* < 0.01, and ^^^*P* < 0.001. *CircPLXNB2 OE* circPLXNB2 overexpression, *circPLXNB2 shRNA* circPLXNB2 short hairpin RNA

**Fig. 6:**
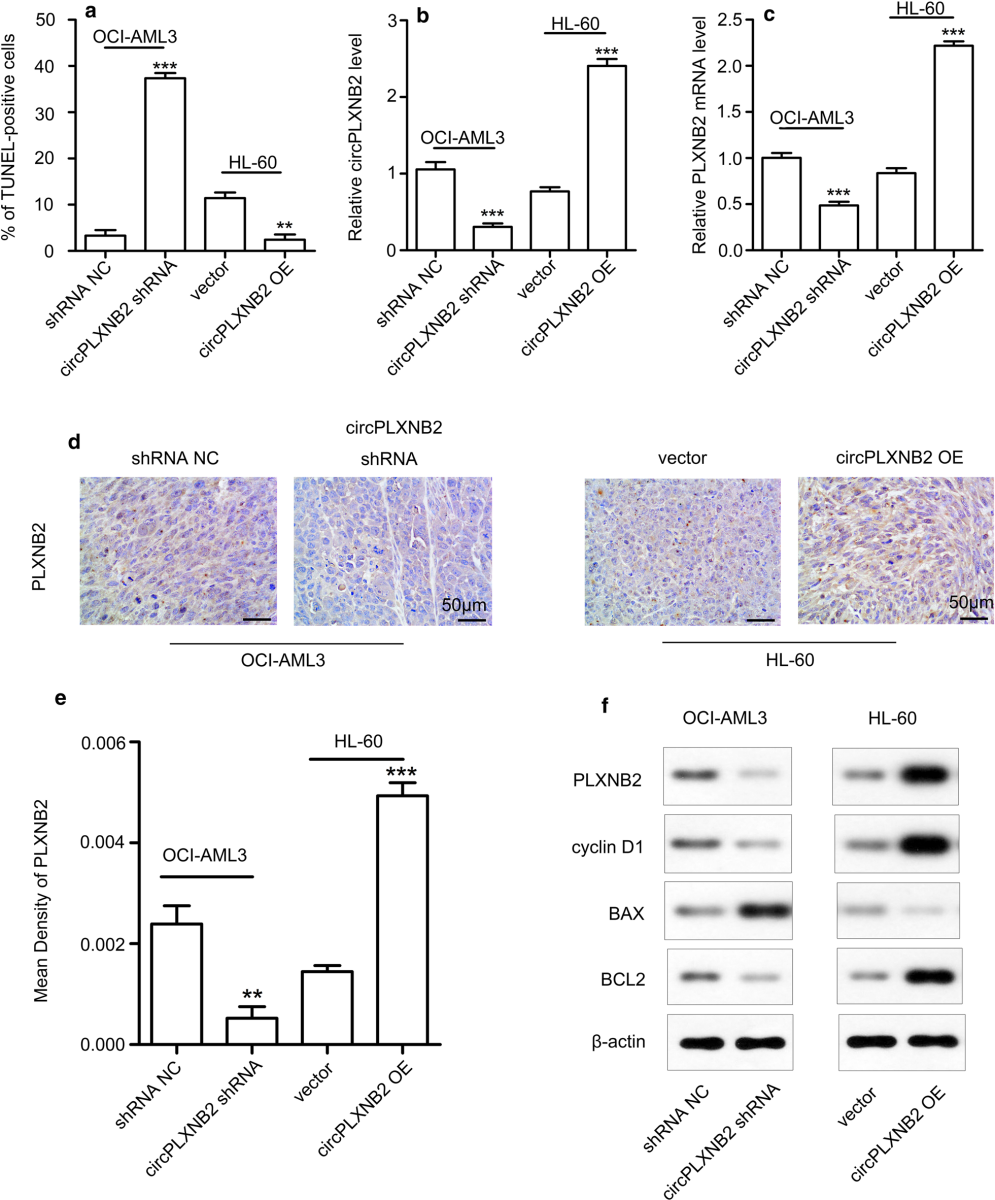
Effect of circPLXNB2 expression on PLXNB2, apoptosis and proteins associated with cell cycle in vivo. **a** TUNEL-positive cells were measured in 3 randomly chosen fields from each biopsy (3 × 10^4^ μm/field). qRT-PCR was used to detect the expression of circPLXNB2 (**b**) and the PLXNB2 mRNA (**c**) in vivo. **d** IHC staining of xenograft tumour tissues obtained from three mice randomly selected from each group (n = 6). Representative IHC images with an original magnification of 400× show the expression of PLXNB2 in different murine AML xenograft models. **e** Image software measured the average optical density of the selected area in sections subjected to IHC staining for PLXNB2. **f** Representative Western blots showing the expression of BCL2, BAX, cyclin D1 and PLXNB2 in different AML xenograft models. Western blotting was performed on the xenograft tumour tissues obtained from three mice randomly selected from each group (n = 6) and β-actin was used as a loading control. The vector or shRNA NC was used as a control. Each experiment was repeated three times. **P < 0.01 and ***P < 0.001. *CircPLXNB2 OE* circPLXNB2 overexpression, *circPLXNB2 shRNA* circPLXNB2 short hairpin RNA

We subcutaneously inoculated AML cells into female NOD/SCID mice to establish xenograft tumour models, explore whether circPLXNB2 promotes tumour growth in vivo. During the course of the 28-day observation period, tumours grew faster in mice inoculated with HL-60 circPLXNB2 OE cells compared with the control, but tumour growth was inhibited in mice inoculated with OCI-AML3 circPLXNB2 shRNA cells (Fig. 5a, b). At the end of 28 days, the tumour weight was significantly higher in mice inoculated with cells expressing circPLXNB2 at high levels (Fig. 5c). We performed H&E staining of tissue derived from AML xenograft model mice to observe the morphologies of tumour cells. The TUNEL technique was used to detect the apoptosis of tumour cells in tissue. As shown in Fig. 5d, compared with the control group, nuclear pyknosis and apoptosis were significantly increased in tumour cells transfected with the circPLXNB2 shRNA. As shown in Fig. 5e, few AML cells overexpressing circPLXNB2 in the tumour tissues from the mouse xenograft model underwent apoptosis. The number of TUNEL-positive cells in mice was significantly increased upon circPLXNB2 shRNA transfection, indicating that the circPLXNB2 shRNA induces apoptosis in vivo (Fig. 6a). In addition, we performed qRT-PCR (Fig. 6b, c), IHC staining (Fig. 6d, e) and Western blotting (Fig. 6f) to analyse the expression of circPLXNB2, the PLXNB2 mRNA and protein, and proteins associated with cell cycle and apoptosis in mouse xenograft tumour tissues. In the mice treated with circPLXNB2 OE cells, the expression of PLXNB2, BCL2 and cyclin D1 was elevated, while the level of BAX decreased. The expression of PLXNB2, BCL2 and cyclin D1 was reduced, while the level of BAX increased in mice treated with cells expressing the circPLXNB2 shRNA. Based on these results, circPLXNB2 promotes tumour cell proliferation, inhibits apoptosis, and increases the leukemic burden in vivo.

## Discussion

In the 1970s, circRNAs were initially discovered [36], and subsequently detected in various organisms, confirming the widespread presence of circRNAs. Notably, circRNAs are highly enriched in some human tissues, such as the brain [37], platelets [38], and haematopoietic progenitor cells that differentiate into lymphoid and myeloid cells [39]. Moreover, due to the high stability and specificity of circRNAs expressed in different diseases or tissues, circRNAs have been suggested to be a potential disease biomarker. In the present study, circPLXNB2 expression was obviously increased in patients with AML, particularly in patients with EMI. The results of the ROC curve analysis suggested that high circPLXNB2 expression might be a potential diagnostic biomarker to distinguish patients with AML from healthy people. An analysis of associations between several clinical features of AML patients and circPLXNB2, as shown in Additional file 2: Table S1, did not reveal distinct differences in these clinical and laboratory characteristics. Moreover, the Kaplan–Meier analysis disclosed that patients with AML and high circPLXNB2 expression experienced obviously shorter OS and LFS. According to the aforementioned results, we concluded that the level of circPLXNB2 expression may be an important indicator to evaluate the prognosis and efficacy of AML. Clearly, more in-depth studies of large numbers of patients with AML are needed to confirm our findings.

Based on the results mentioned above, circPLXNB2 is likely to play a cancer-promoting role in AML. We conducted a series of experiments to observe the effect of circPLXNB2 on AML cells in vitro and *vivo*. In the current study, circPLXNB2 promoted the proliferation and migration of AML cells, but inhibited apoptosis. Conversely, knockdown of circPLXNB2 increased apoptosis and inhibited the proliferation and migration of AML cells. Moreover, circPLXNB2 promoted tumour growth and inhibited apoptosis in vivo. Thus, circPLXNB2 plays a cancer-promoting role in AML.

The cytoplasmic segment of PLXNB2 contains a GTP kinase domain that regulates the downstream Rho A pathway and subsequently activates MAPK, PI3K/AKT and other signalling pathways to promote cell proliferation and differentiation [22, 40]. PLXNB2 also plays a critical role in invasive growth and cell migration [41]. In our previous study analysing data from The Cancer Genome Atlas database, PLXNB2 was associated with poor prognosis for patients with AML, and was likely to be involved in the regulation of leukaemia cell infiltration [21]. Remarkably, in this study, a positive correlation was observed between the expression levels of circPLXNB2 and PLXNB2 in patients with AML. Recent studies have confirmed that the subcellular localization and distribution of circRNAs may be closely related to their functions: some circRNAs are mainly distributed in the cytoplasm and likely to participate in post-transcriptional regulation [13], other circRNAs are mainly distributed in the nucleus and may regulate the expression of parental genes through special RNA-RNA interactions [12], some of these circRNAs that are mainly distributed in the nucleus interact with RNA-binding proteins to regulate gene transcription [42]. According to the RNA-FISH analysis, circPLXNB2 is mainly located in the nucleus, suggesting that this circRNA is likely to regulate the transcription of its parental gene PLXNB2. Then, by performing in vitro and in vivo experiments, we observed that overexpression of circPLXNB2 up-regulates the expression of the PLXNB2 mRNA and protein, while knockdown of circPLXNB2 obviously inhibited PLXNB2 expression. Based on our research results mentioned above, we boldly speculate that circPLXNB2, an oncogene, may promote the progression of AML by up-regulating the expression of PLXNB2, but further functional studies are needed to confirm our hypothesis.

However, there are limitations in this study. We plan to compare the expression level of circPLXNB2 in paired AML samples obtained at the initial diagnosis and relapse with matched AML patient samples obtained after complete remission, and a larger sample size is needed to define circPLXNB2 as ideal biomarker. In this manuscript, we found that the up-regulation of circPLXNB2 increased PLXNB2 expression. However, it was found that circPLXNB2 expression was low and the expression level of PLXNB2 mRNA was high in THP1 and MV4;11 cells. We analysed that this phenomenon might be related to the complex network of regulating gene expression. The previous studies have confirmed that the expression of PLXNB2 is regulated by miR-126-3p, TNF-α and other factors [27, 29, 43]. And circPLXNB2 is also found to have an effect on the expression of PLXNB2 in this manuscript. These results indicate that the network regulating the expression of PLXNB2 is complex. Based on the comprehensive analysis of this study, we can conclude that the expression of PLXNB2 is affected by many factors, and circPLXNB2 is one of the important factors affecting the expression of PLXNB2. And whether up-regulated PLXNB2 can promote the production of circPLXNB2 and generate positive feedback is still unclear. In the future, we will also design further functional experiments to explore and verify the regulatory relationship between circPLXNB2 and PLXNB2, and clarify the regulatory mechanism. Meanwhile, the mechanism underlying the effects of circPLXNB2 on the microenvironment or leukemic niche remains to be further clarified. In addition, in the present study, we only established a subcutaneous AML tumour model in mice to facilitate the observation of tumour growth and the acquisition of tumour tissues. Next, we will design and establish AML patient-derived xenograft mice that more closely resemble the pathogenesis of AML to better study the roles of circPLXNB2 and PLXNB2 in the process of AML in vivo.

## Conclusion

In summary, we validated the high expression of circPLXNB2 in patients with AML, particularly in patients with EMI. Elevated circPLXNB2 levels were associated with poor clinical outcomes in patients with AML. Our results confirmed a positive relationship between the expression levels of circPLXNB2 and PLXNB2 in AML, both of which were up-regulated. CircPLXNB2 plays an important role in promoting the proliferation and inhibiting the apoptosis of AML cells probably by regulating the expression of PLXNB2. Knockdown of circPLXNB2 significantly reverses its oncogenic effects. The results from our study highlight the potential of circPLXNB2 as a new prognostic predictor and therapeutic target for AML in the future.

## Supplementary Information

**Additional file 1: Figure S1.** The distribution of circPLXNB2 expression in AML patients.

**Additional file 2: Table S1.** Comparison of clinical manifestations and laboratory features between AML patients with low and high expression of circPLXNB2.

**Additional file 3: Figure S2.** Rescuing the expression of circPLXNB2 in OCI-AML3 cells which had circPLXNB2 silenced.

## Data Availability

The data described in this manuscript are contained in published articles or available from the corresponding author upon reasonable request.

## Abbreviations

AML: Acute myeloid leukaemia
BM: Bone marrow
EMI: Extramedullary infiltration
CircRNAs: Circular RNAs
FAB: French–American–British
BMMCs: Bone marrow mononuclear cells
circPLXNB2 OE: CircPLXNB2 overexpression
circPLXNB2 shRNA: CircPLXNB2 short hairpin RNA
qRT-PCR: Real‐time quantitative polymerase chain reaction
GAPDH: Glyceraldehyde 3-phosphate dehydrogenase
cDNA: Complementary DNA
RNA-FISH: RNA fluorescence in situ hybridization
DAPI: 4,6-Diamidino-2-phenylindole
CCK-8: Cell Counting Kit-8
PI: Propidium iodide
FBS: Foetal bovine serum
BCL2: B-cell lymphoma-2
BAX: BCL2-associated X protein
NC: Negative control
H&E: Haematoxylin and eosin
ANOVA: One-way analysis of variance
ROC: Receiver operating characteristic
AUC: Area under the ROC curve
IHC: Immunohistochemical
gDNA: Genomic DNA
FCM: Flow cytometry
OS: Overall survival
LFS: Leukaemia-free survival
