# Supplementary material for: A circular RNA derived from PLXNB2 as a valuable predictor of the prognosis of patients with acute myeloid leukaemia
Source: J Transl Med. 2021 Mar 23;19:123. doi: 10.1186/s12967-021-02793-7 (PMC7988933; doi:10.1186/s12967-021-02793-7)
Supplement: Supplementary file 1 — Additional file 1: Figure S1. The distribution of circPLXNB2 expression in AML patients. [file 12967_2021_2793_MOESM1_ESM.docx]

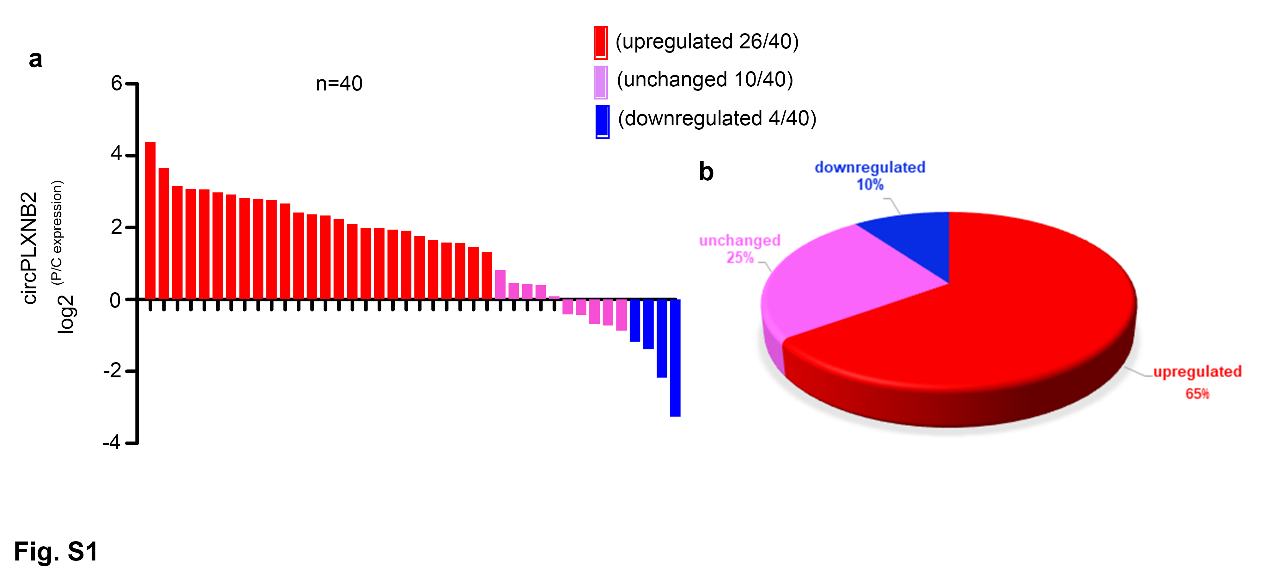


**Figure S1.** The distribution of circPLXNB2 expression in AML patients. Histogram (**a**) and pie (**b**) chart of proportions of AML samples in which circPLXNB2 expression was upregulated (26/40, 65%, red), downregulated (4/40, 10%, blue), or no change (10/40, 25%, purple). Log2 ^(P/C expression)^ value >1 as significantly higher expression, which <-1 as lower expression, and between -1 and 1 as no significant change. *P* AML patient, *C* healthy control.
