# Supplementary material for: A circular RNA derived from PLXNB2 as a valuable predictor of the prognosis of patients with acute myeloid leukaemia
Source: J Transl Med. 2021 Mar 23;19:123. doi: 10.1186/s12967-021-02793-7 (PMC7988933; doi:10.1186/s12967-021-02793-7)
Supplement: Supplementary file 2 — Additional file 2: Table S1. Comparison of clinical manifestations and laboratory features between AML patients with low and high expression of circPLXNB2. [file 12967_2021_2793_MOESM2_ESM.docx]

**Additional Materials**

**Table S1** **Comparison of clinical manifestations and laboratory features between AML patients with low and high expression**

|  | **CircPLXNB2 expression** | |  |
| --- | --- | --- | --- |
| **Patient’s parameters** | **circPLXNB2^Low^ (n=20)** | **circPLXNB2^High^ (n=20)** | ***P* Value** |
| **Sex (male/female)** | 9/11 | 13/7 | 0.2036 |
| **Median age (range), years** | 43 (17-71) | 34 (18-76) | 0.1983 |
| **Median WBC (range), ×10^9^/L** | 14.98 (2.48-267.6) | 45.75 (1.01-198.1) | 0.4230 |
| **Median hemoglobin (range), g/L** | 81.45(30.53-130) | 83.82(42.34-113.8) | 0.3188 |
| **Median platelets (range), ×10^9^/L** | 42.19(8.1-245) | 34.89(9-95.56) | 0.2160 |
| **FAB** |  |  | 0.4665 |
| M1 | 2 (13%) | 4 (10%) |  |
| M2 | 6 (13%) | 5 (13%) |  |
| M4 | 5 (10%) | 5 (13%) |  |
| M5 | 7 (5%) | 4 (10%) |  |
| M6 | 0 (0%) | 2 (5%) |  |
| **Karyotype classification** |  |  | 0.1102 |
| Favorable | 6 (15%) | 7 (18%) |  |
| Intermediate | 8 (20%) | 11 (28%) |  |
| Poor | 7 (18%) | 1 (3%) |  |
| No data | 0 (0%) | 1 (3%) |  |
| **Karyotype** |  |  | 0.7922 |
| Normal | 7 (18%) | 11 (28%) |  |
| t (8;21) | 4 (10%) | 4 (10%) |  |
| inv (16) | 2 (5%) | 3 (8%) |  |
| Complex | 1 (3%) | 1 (3%) |  |
| Others | 6 (15%) | 0 (0%) |  |
| No data | 0 (0%) | 1 (3%) |  |
| **Gene mutation** |  |  |  |
| FLT3-ITD (+/-) | 6 /13 | 8/9 | 0.4955 |
| NPM1 (+/-) | 6/13 | 4/14 | 0.7140 |
| CEBPA (+/-) | 8/11 | 9/9 | 0.6301 |
| RUNX1 (+/-) | 5/14 | 2/16 | 0.4048 |
| IDH1/2 (+/-) | 4/15 | 5/16 | 0.8348 |
| DNMT3A (+/-) | 4/14 | 3/15 | 0.6737 |

*AML* acute myeloid leukemia, *WBC* white blood cells, *FAB* French-American-British classification. Cut-off 0.00148, defined as the median for all patients with AML.
