# Supplementary material for: A circular RNA derived from PLXNB2 as a valuable predictor of the prognosis of patients with acute myeloid leukaemia
Source: J Transl Med. 2021 Mar 23;19:123. doi: 10.1186/s12967-021-02793-7 (PMC7988933; doi:10.1186/s12967-021-02793-7)
Supplement: Supplementary file 3 — Additional file 3: Figure S2. Rescuing the expression of circPLXNB2 in OCI-AML3 cells which had circPLXNB2 silenced. [file 12967_2021_2793_MOESM3_ESM.docx]

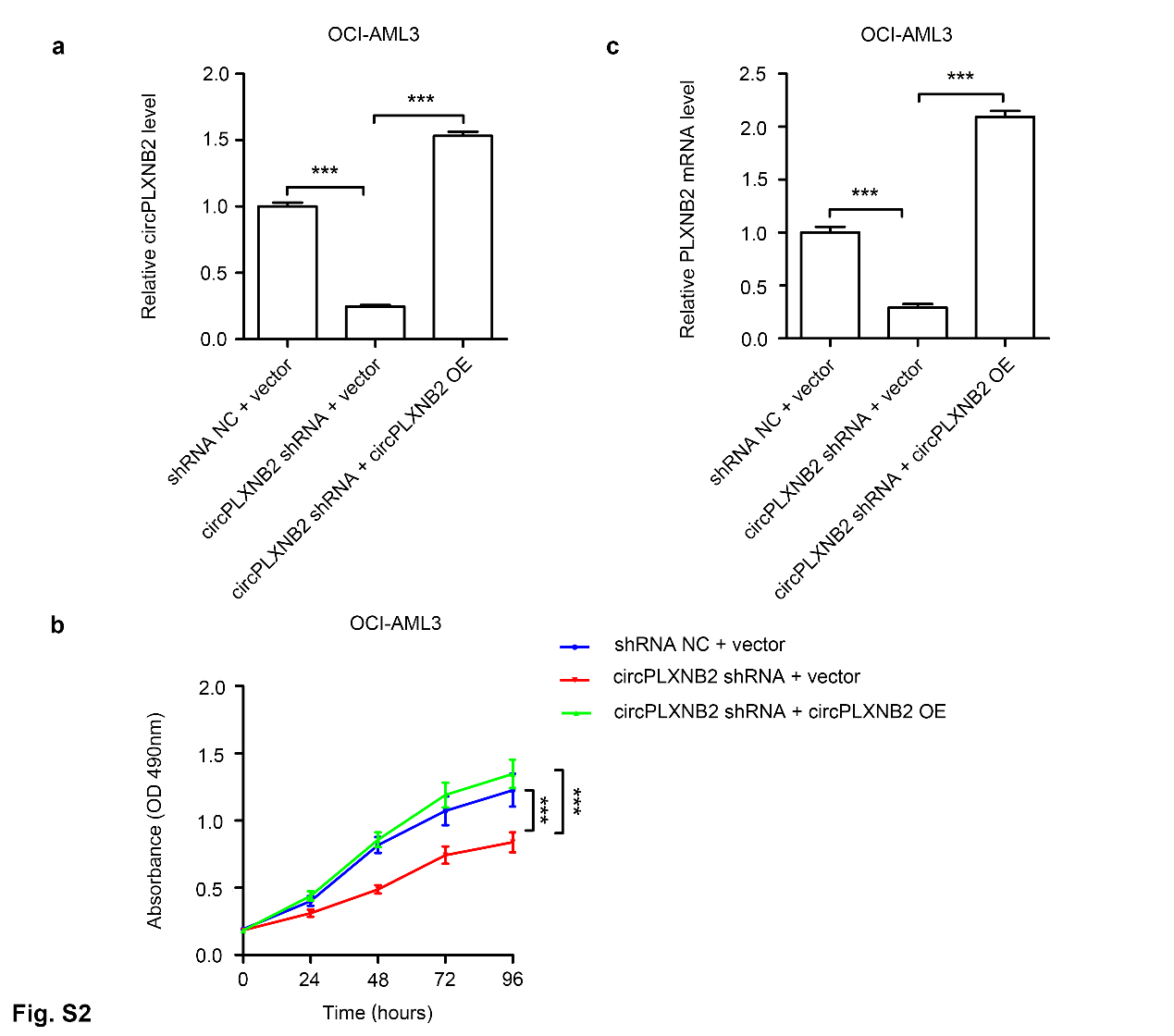


**Figure S2.** Rescuing the expression of circPLXNB2 in OCI-AML3 cells which had circPLXNB2 silenced. The expression of circPLXNB2 (**a**) and the PLXNB2 mRNA (**c**) was detected using qRT-PCR. **b** Cell proliferation was measured with the CCK-8 method. Each experiment was repeated three times. ****P* < 0.001. *CircPLXNB2 OE* circPLXNB2 overexpression, *circPLXNB2 shRNA* circPLXNB2 short hairpin RNA, *NC* negative control.
